# Synthesis and Characterization of Tungsten Suboxide W_n_O_3n−1_ Nanotiles

**DOI:** 10.3390/nano11081985

**Published:** 2021-08-02

**Authors:** Luka Pirker, Bojana Višić, Janez Kovač, Srečo D. Škapin, Maja Remškar

**Affiliations:** 1 Jožef Stefan Institute, Jamova Cesta 39, 1000 Ljubljana, Slovenia; luka.pirker@ijs.si (L.P.); janez.kovac@ijs.si (J.K.); sreco.skapin@ijs.si (S.D.Š.); maja.remskar@ijs.si (M.R.); 2 Institute of Physics Belgrade, University of Belgrade, Pregrevica 118, 11080 Belgrade, Serbia; 3 Faculty for Mathematics and Physics, University of Ljubljana, Jadranska Ulica 19, 1000 Ljubljana, Slovenia

**Keywords:** tungsten oxides, nanotiles, nanomaterials

## Abstract

W_n_O_3n−1_ nanotiles, with multiple stoichiometries within one nanotile, were synthesized via the chemical vapour transport method. They grow along the [010] crystallographic axis, with the thickness ranging from a few tens to a few hundreds of nm, with the lateral size up to several µm. Distinct surface corrugations, up to a few 10 nm deep appear during growth. The {102}_r_ crystallographic shear planes indicate the W_n_O_3n−1_ stoichiometries. Within a single nanotile, six stoichiometries were detected, namely W_16_O_47_ (WO_2.938_), W_15_O_44_ (WO_2.933_), W_14_O_41_ (WO_2.928_), W_13_O_38_ (WO_2.923_), W_12_O_35_ (WO_2.917_), and W_11_O_32_ (WO_2.909_), with the last three never being reported before. The existence of oxygen vacancies within the crystallographic shear planes resulted in the observed non-zero density of states at the Fermi energy.

## 1. Introduction

In the family of transition metal oxide materials, semiconducting WO_3_ is among the most studied, due to its promising practical applications. It has already been successfully used as a catalyst for water splitting [1], in gas/temperature sensors [2,3], in optoelectronics [4], or as a component in supercapacitors [5]. The crystal structure of WO_3_ is usually described in terms of corner-sharing WO_6_ octahedra. The structure can differ from the ideal cubic ReO_3_ type structure due to different tilting angles, displacement of the W cation, and rotation of WO_6_ octahedra. Its phase transitions have been thoroughly studied [6,7,8,9], and various nanometre-sized particles, nanowires and flakes were synthesized [10,11].

The sub-stoichiometric tungsten oxide phases, WO_3−x_, with 0 < x < 1, provide the opportunity to synthesize and study nanoparticles with great variety of shapes, sizes and physical properties. According to the literature, for x ≤ 0.2, the WO_3−x_ crystallize into phases with the chemical formula W_n_O_3n−1_ or W_n_O_3n−2_, which are often referred to as Magnéli phases [12,13]. The W_n_O_3n−1_ stoichiometry crystallizes in the P2/a symmetry with a monoclinic unit cell containing two W_n_O_3n−1_ moieties, while the W_n_O_3n−2_ crystallizes in the P2/m symmetry in a monoclinic unit cell with one W_n_O_3n−2_ moiety. The oxygen deficiency present in WO_3−x_ is compensated with the formation of crystallographic shear (CS) planes, where some of the corner-sharing WO_6_ octahedra become edge-sharing. In W_n_O_3n−1_ structures, four WO_6_ octahedra are joined by edges, while in W_n_O_3n−2_ the number of these octahedra is six. With further reduction (x > 0.2), edge and face-sharing WO_6_ octahedra emerge, forming pentagonal columns and hexagonal tunnels [14]. The crystal structures of these materials are found to be orthorhombic for W_32_O_84_ and W_3_O_8_, monoclinic for W_18_O_49_, W_17_O_47_, W_20_O_58_, and W_25_O_73_, and tetragonal for W_5_O_14_ [15]. These varieties stem from different oxygen deficiencies within the nanostructures [16,17].

Here, we report on new tungsten suboxide nanostructures crystallized in the form of nanotiles. The nanotiles are composed of different W_n_O_3n−1_ stoichiometries, three of which were observed for the first time. High-resolution transmission electron microscopy (HRTEM), scanning electron miscroscopy (SEM), X-ray diffraction (XRD), Raman spectroscopy, X-ray photoelectron spectroscopy (XPS) and atomic force microscopy (AFM) were used to characterize the nanotiles.

## 2. Materials and Methods

### 2.1. Synthesis

The nanotiles were synthesized via the chemical vapour transport reaction (CVT). Iodine was used as the transport agent and nickel as the growth promoter. Quartz ampules were filled with 352.7 mg of WO_3_ powder (Sigma-Aldrich, St. Louis, MO, USA, 99.99%), 37.5 mg of nickel (metal foil) and 562 mg of iodine (1–3 mm beads, Sigma-Aldrich, St. Louis, MO, USA, 99.7%). Ampules were evacuated down to 10^−5^ mbar, and the transport reaction was running for 500 h. The material was transported from hot zone of the furnace (1133K) to the growth zone (1009K).

### 2.2. X-ray Diffraction 

X-ray diffraction (XRD) was performed using a D4 Endeavor diffractometer (Bruker AXS GmbH, Karlsruhe, Germany) at room temperature. A quartz monochromator Cu Ka1 radiation source (λ = 0.1541 nm) and a Sol-X energy dispersive detector were used. The angular range (2ϑ) was in the range from 10° to 70°, with a step size of 0.02° and collection time of 4 s.

### 2.3. Raman Spectroscopy

Raman spectra of the nanotiles were recorded by an Alpha300 R (WITec, Ulm, Germany) confocal Raman imaging system. Measurements were performed in backscattered geometry using a frequency doubled Nd:YAG laser (532 nm). The laser power was kept under 5 mW for standard measurements (to prevent oxidation and damage of the material). For laser power dependence studies, the power was varied from 0.06 mW to 24.7 mW. The sample was dispersed in ethanol and drop-casted on a chromium plate as a substrate with a featureless Raman spectrum.

### 2.4. Scanning Electron Microscopy

Scanning electron microscopy (SEM) images and cross-sections of the samples for TEM analysis were obtained using a Helios NanoLab 650 (Thermo Fisher, Waltham, MA, USA) Focused Ion Beam-scanning electron microscope (FIB). The nanotiles were drop-casted on a silicon wafer for SEM studies.

### 2.5. High-Resolution Transmission Electron Microscopy and Electron Diffraction

High-resolution transmission electron microscopy (HRTEM) and electron diffraction (ED) images were acquired using a Cs probe-corrected TEM/STEM JEOL ARM 200CF (JEOL, Peabody, MA, USA) microscope equipped with a cold-FEG electron source, operating at 200 kV. Distances between atomic columns and angles between their rows were measured using Digital Micrograph software. An accuracy of 0.04 Å in distance and 0.5° in angle was achieved. All HRTEM images were filtered using the Average Background Subtraction Filter method described in [18].

### 2.6. Atomic Force Microscopy

Atomic force microscopy (AFM) in contact mode was performed with an Omicron UHV VT-AFM (Scienta Omicron, Taunusstein, Germany) operating at 10^−9^ mbar. Silicon Cantilevers CSG10 (NT-MDT, Moscow, Russia) with a typical force constant of 0.11 N/m were used.

### 2.7. X-ray Photoelectron Spectroscopy

X-ray photoelectron spectroscopy XPS analysis was carried out on the PHI-TFA XPS spectrometer produced by Physical Electronics, Chanhassen, MN, USA. Samples were mounted on the metallic sample holder and introduced in ultra-high vacuum spectrometer. The vacuum during the XPS analyses was in the range of 10^−9^ mbar. The analysed area was 0.4 mm in diameter and the analysed depth was about 3–5 nm. Sample surfaces were excited by X-ray radiation from monochromatic Al source at photon energy of 1486.6 eV. The high-energy resolution spectra were acquired with energy analyser operating at resolution of about 0.6 eV and pass energy of 29 eV. The accuracy of binding energies was about ±0.2 eV. Three places on every sample were analysed. High resolution spectra were fitted with Gauss-Lorentz functions and Shirley function was used for background removal. For the XPS measurements, the ethanol suspension of nanotiles was deposited on an oxidized Si wafer, dried at room temperature and inserted into ultra-high vacuum of the spectrometer.

## 3. Results

### 3.1. Electron Microscopy 

**Figure 1:**
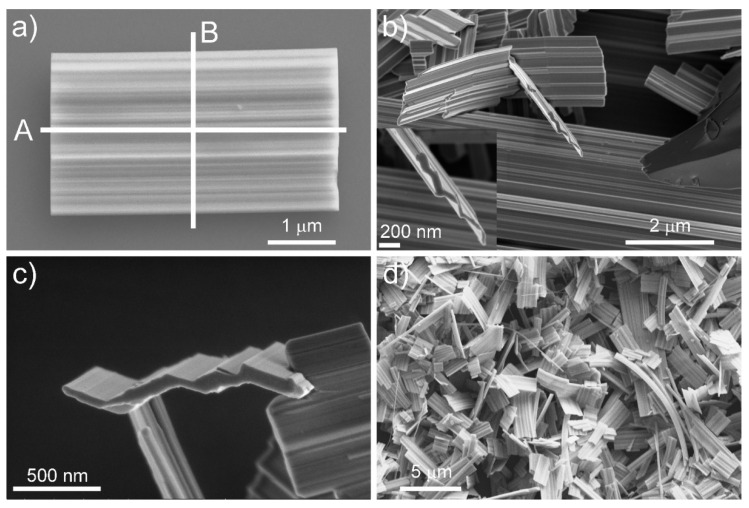
(**a**) A single nanotile, with lines A and B representing the direction of the cross-sections for the TEM lamellas; (**b**–**d**) SEM images of different nanotiles.

**Figure 2:**
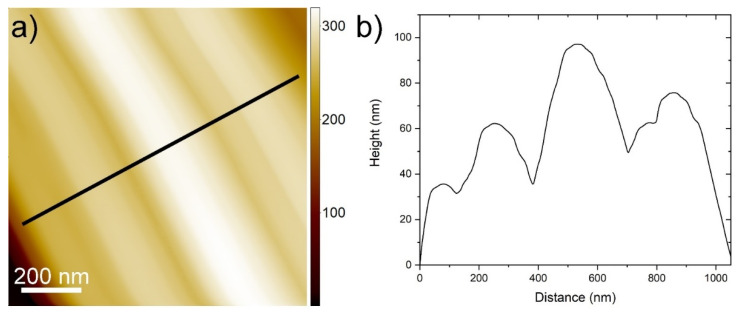
(**a**) An AFM image of a nanotile with a line profile showing corrugations in (**b**).

The nanotiles, depicted in Figure 1, grow on the ampule walls in the form of a blue powder. A single nanotile usually grows in a rectangular shape, a few micrometres in width and up to 10 µm in length, as shown in Figure 1. The thickness of the nanotiles varies from a few 10 nm up to a few 100 nm, as seen in Figure S1 and Figure 1. They have distinct corrugations, which can be up to a few 10 nm deep, as seen in Figure 2. To determine the structure of the nanotiles, two cross-section lamellas perpendicular and parallel to the corrugations were prepared for further TEM analysis, as shown in Figure 1a.

**Figure 3:**
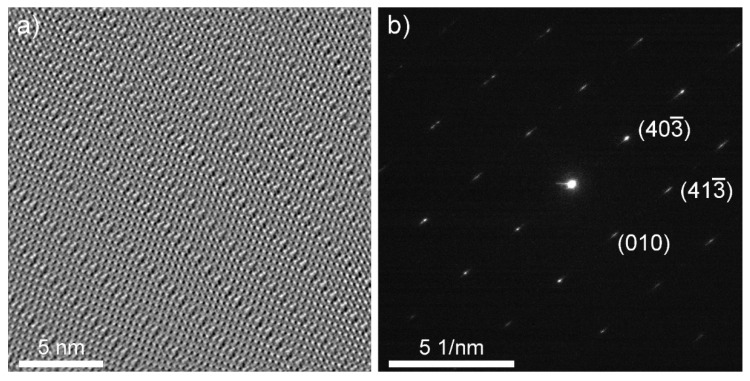
(**a**) HRTEM image along the [010] direction (lamella B). (**b**) An electron diffraction of the [304] zone (lamella A).

**Figure 4:**
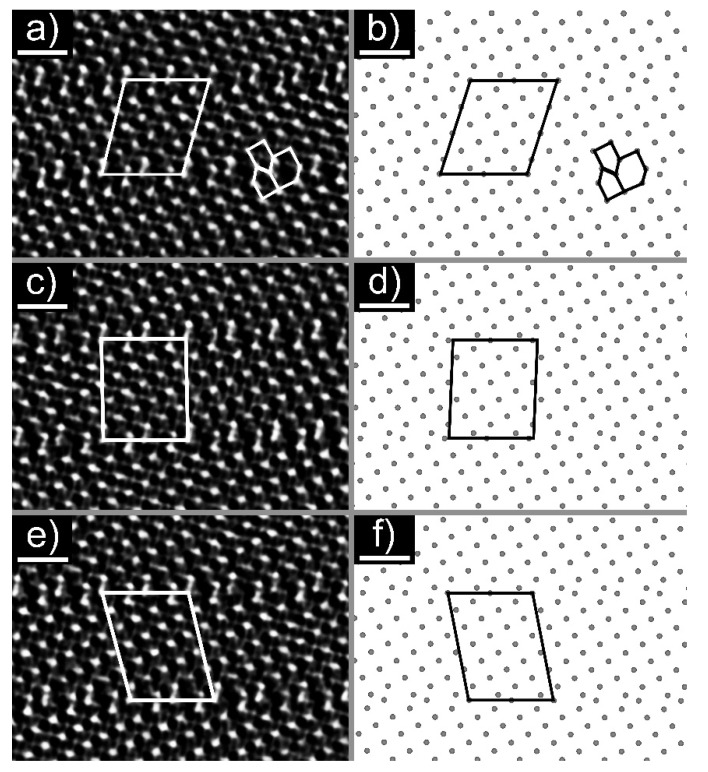
HRTEM images of: (**a**) W_11_O_32_, (**c**) W_12_O_35_, and (**e**) W_13_O_38_ with the proposed unit cell. The scale bar is 1 nm. Simulated structures of: (**b**) W_11_O_32_, (**d**) W_12_O_35_, and (**f**) W_13_O_38_ with the proposed unit cell.

An HRTEM image of the cross-section lamella B is shown in Figure 3a and Figure S2. Figure 3a was taken along the [010] direction, and was used to determine the stoichiometry of the nanotiles. The parallel contrast lines are crystallographic shear (CS) planes, which are characteristic for W_n_O_3n−1_ and W_n_O_3n−2_ phases. Only {102}_r_ CS planes were observed, indicating that only W_n_O_3n−1_ structures grow inside the nanotiles [12,19]. Six stoichiometries were determined by measuring the unit cell parameters *a*, *c,* and β: W_16_O_47_ (WO_2.938_), W_15_O_44_ (WO_2.933_), W_14_O_41_ (WO_2.928_), W_13_O_38_ (WO_2.923_), W_12_O_35_ (WO_2.917_), and W_11_O_32_ (WO_2.909_), of which the last three were not experimentally observed to date [20]. The unit cell parameters *a*, *c,* and β of the observed phases are presented in Table 1. The *a* axis is oriented along the CS planes, while the *c* axis is directed towards the CS plane at the angle β, relative to axis *a*. An electron diffraction was performed on lamella A, Figure 3b. The reflections (010), $\left(40\bar{3}\right)$ and $\left(41\bar{3}\right)$ correspond to interlayer distances of 3.79 Å, 3.56 Å, and 2.61 Å, respectively. The first estimation of the unit cell parameter *b* was determined from the (010) reflection with the value of 3.79 Å. The second value for *b* (3.86 Å) was determined from the average distance between the tungsten atoms that are not part of the CS planes. The mean value of the unit cell parameter *b* is 3.83 Å. The theoretical tungsten atom positions and unit cell parameters for the newly observed phases were calculated using the model proposed in ref. [12]. The parameters d and e used in the model were determined from the HRTEM and electron diffraction images and are schematically shown in Figure S3: (i) interatomic distance between tungsten atoms that are not part of the CS plane that should equal unit cell parameter *b* (d = 3.83 Å); (ii) interatomic distance between tungsten atoms that are part of the CS plane, where the tungsten octahedra are joined by edges (e = 2.92 Å). The experimental unit cell parameters are in good agreement with the calculated ones. The unit cells are schematically drawn on the HRTEM and simulated structure images and are shown in Figure 4.

### 3.2. X-ray Diffraction

**Figure 5:**
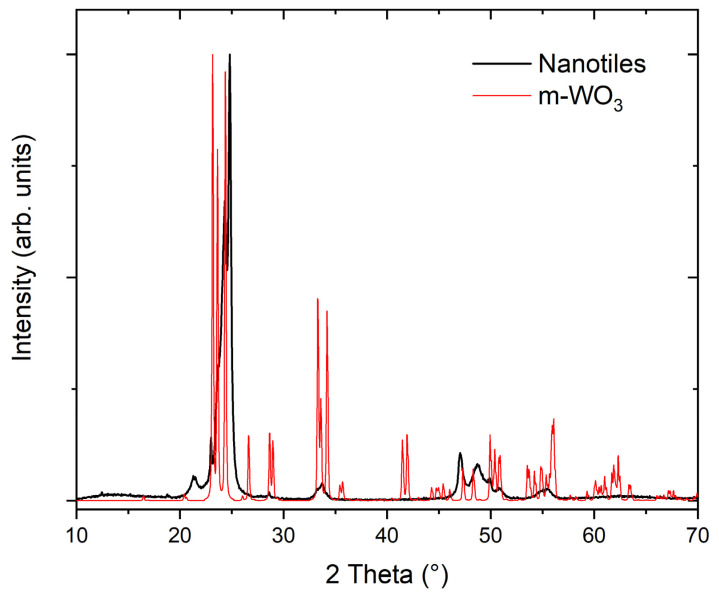
XRD pattern of the WO_3−x_ nanotiles and of m-WO_3_ (PDF2: 01-072-1465).

The XRD pattern of the nanotiles is shown in Figure 5. Due to their multi-stoichiometric structure, the XRD pattern is composed of diffraction lines corresponding to all tungsten suboxide phases present in the nanotiles. The low-angle diffraction lines were used to determine the most prominent phase, as they are different for each stoichiometry and do not overlap with the m-WO_3_ phase. The measured diffractogram had the best match with the W_14_O_41_ (WO_2.928_) stoichiometry, indicating that this is the phase the majority of the nanotiles crystallize in. The position of the diffraction lines, their relative intensities and the assigned (hkl) indices, are presented in Table 2. Additionally, the (010) line closely matches with the *b* unit cell parameter obtained from the HRTEM images. In Figure 5, the measured XRD pattern is compared to the m-WO_3_ one (PDF2: 01-072-1465).

### 3.3. X-ray Photoelectron Spectroscopy

**Figure 6:**
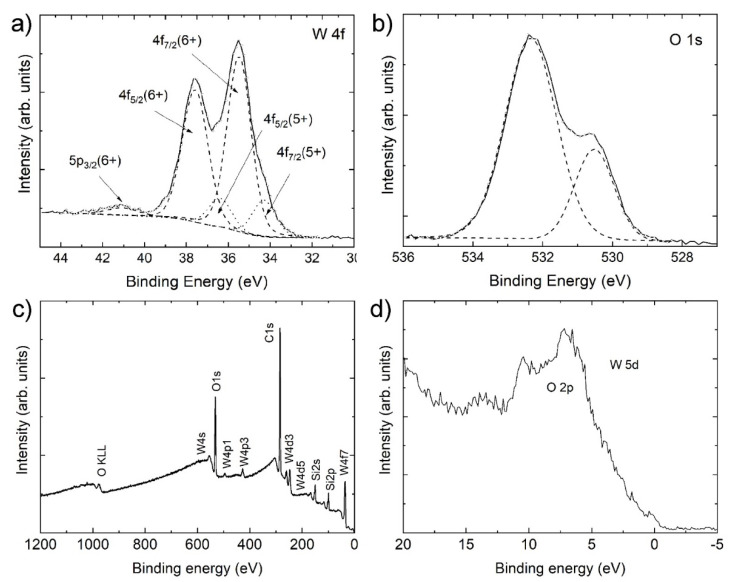
XPS spectra of the nanotiles: (**a**) the W 4f spectrum; (**b**) the O 1s spectrum; (**c**) XPS survey spectrum; and (**d**) the valence band spectrum.

Figure 6 shows the W 4f and O 2p spectra, the survey spectrum, and the valence band spectrum of the nanotiles. The energy distribution of W 4f core levels is presented in Figure 6a. The spectrum can be deconvoluted into two doublets, with the additional fifth component (around 41.1 eV) corresponding to the W 5p photoelectrons. The main peaks, representing 84% of total W 4f, appear at 35.5 and 37.6 eV, corresponding to 4f_7/2_ and 4f_5/2_, respectively, of W in 6+ oxidation state [21]. The remaining 16% are attributed to a doublet positioned at 34.3 and 36.4 eV of the 4f_7/2_ and 4f_5/2_ of W in 5+ oxidation states [22,23]. We can disregard the presence of WO_2_ in the nanotiles, as there are no peaks corresponding to 4 + oxidation states (doublets at 33.3 and 35.5 eV) or metallic tungsten (31.2 and 33.4 eV) [24,25].

The oxygen O 1s spectrum can be deconvoluted into two peaks, as shown in Figure 6b. Peak at 530.5 eV, attributing 26% to the O 1s photoelectrons, corresponds to O^2−^ bonded to W^6+^ in WO_3_ [26]. Peak at 532.3 eV may correspond to oxygen O^2−^ bonded to SiO_2_ (substrate), C-O bonds or lower oxidation states of O in W-O bonds. The survey spectrum presented in Figure 6c shows no impurities other than carbon, while the silicon peaks arise due to the SiO_2_ substrate. The valence band spectrum presented in Figure 6d shows a broad O 2p peak with non-negligible density of states at the Fermi energy.

### 3.4. Raman Spectroscopy

**Figure 7:**
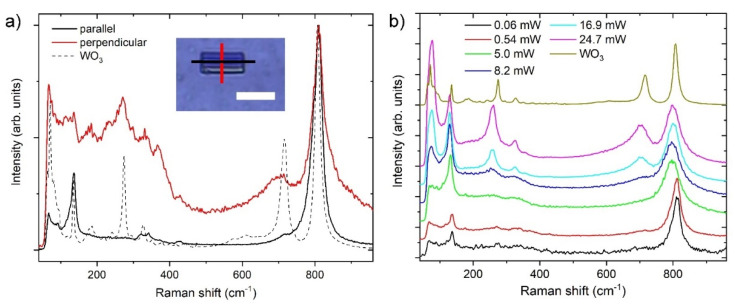
(**a**) Raman spectra of the WO_3−x_ nanotiles taken parallel and perpendicular to the corrugations, and of m-WO_3_ (dashed line). Inset shows an optical image of the nanotile with the red and black line representing the direction of the laser polarisation. (**b**) Power dependency of the Raman spectra.

Raman spectra of the nanotiles are shown in Figure 7a. The spectra were taken with the laser polarisation parallel and perpendicular to the corrugations (i.e., *b* axis). The peak positions and their normalized intensities are presented in Table 3. The Raman spectrum of the nanotiles with the polarisation parallel to the *b* axis reveals six peaks at 136.5, 322.5, 341.5, 426.5, 722, and 810 cm^−1^. The peak at 136.5 cm^−1^ is attributed to the relative translational or rotational motions of WO_6_ octahedral units in the same unit cell (lattice modes), the 322.5, 341.5, and 426.5 peaks to the W-O-W bending modes, while the 722 and 810 cm^−1^ peaks are attributed to the W-O stretching modes [7,27]. On the other hand, Raman spectrum of the nanotiles with the polarisation perpendicular to the *b* axis has nine peaks at 136, 180, 232.5, 271.5, 331.5, 367, 428, 702, and 810 cm^−1^. In both cases the 810 cm^−1^ peak is the most intense one. The dependency of the Raman spectra on orientation is a direct evidence of material anisotropy. Similarly to the previously reported spectra, [20] the spectrum where the polarisation is parallel to the *b* axis has sharper and more pronounced peaks, pointing to a crystal structure with fewer defects and a higher number of W-O bonds with well-defined lengths. The Raman spectrum recorded with the polarisation perpendicularly to the *b* axis has a greater number of peaks in the lattice (<200 cm^−1^) and bending mode (200–400 cm^−1^) region, while the peaks associated with W-O stretching modes (600–900 cm^−1^) are broader, indicating that multiple bond lengths are present [27,28]. The spectra of the nanotiles are compared with the precursor WO_3_ powder, with the most prominent peaks at 72, 135, 273, 372, 716, and 807 cm^−1^. These peaks match the monoclinic γ-phase with the space group P^2^_1_/n, and the total of 48 Raman active modes [29]. Compared with the m-WO_3_ spectrum, the most intense peak at 810 cm^−1^ is slightly red-shifted towards longer wavelengths with regard to the 807 cm^−1^ in m-WO_3_, indicating slightly shorter bonds [7,30]. The peaks at 702 and 428 cm^−1^ are blue-shifted, indicating slightly longer bonds (i.e., shorter wavelengths) compared to the m-WO_3_ peaks situated at 715 cm^−1^ and 434 cm^−1^, respectively.

As these materials tend to be oxidized or damaged under the laser irradiation in ambient conditions, a stepwise laser power dependency measurement was performed. The powers at which the sample underwent change and damage can be easily inferred from the spectra shown in Figure 7b. The spectra in the power range between 0.06 and 0.54 mW are indistinguishable, while at 5 mW the 810 cm^−1^ peak shifts to 798 cm^−1^ and becomes broader, the 136 cm^−1^ peak becomes more intense and shifts to 130 cm^−1^, and the shoulders between 200 and 400 cm^−1^ become more prominent. At this point the sample remains visually undamaged, as concluded from its optical image. The power of 8.2 mW marks the start of the sample damage. This is accompanied with the peak at 130 cm^−1^ becoming the most prominent, the appearance of a new peak at 73 cm^−1^, and a shoulder appears around 710 cm^−1^. At 16.9 mW, the spectrum becomes very similar to that of the WO_3_ precursor, marking the complete oxidation of the nanotile due to the heating in ambient oxygen. This is evidenced by a clear appearance of the peak at 702 cm^−1^, albeit blue-shifted and broader (716 cm^−1^ for m-WO_3_). The two shoulders between 200 and 400 cm^−1^ transform into peaks at 258 and 325 cm^−1^ (274 and 327 cm^−1^ for m-WO_3_). Additionally, the peak at 76 cm^−1^ becomes the most intense one. For higher laser powers, no other new peaks appear.

## 4. Discussion

The reported nanotiles are composed of multiple W_n_O_3n−1_ phases, with three of those not observed to date. As previously reported, the multiphase nature of a single nanotile could stabilize the W_n_O_3n−1_ phases [20]. In our previous report, the similar multi-stoichiometric platelets had a flat, corrugation-free surface, while the nanotiles have distinct corrugations with tens of nm in depth. The change in the morphology could be explained with a slightly different overall stoichiometry. Another reason for this change may be because the nanotiles did not have a template from which to grow, while the platelets grew epitaxially from a nanowire [20]. Similar corrugations are also present in other tungsten suboxides [31,32,33] and could contribute to the stabilization of different phases. It is presumed that the nanotiles grow faster along the [010] crystallographic axis (along the corrugations), as the length of the nanotiles varies, while the width is remaining quite uniform.

The XRD pattern of the nanotiles differs from a typical XRD pattern of the m-WO_3_ especially in the low-angle region. Due to the P2/a symmetry of the W_n_O_3n−1_ stoichiometries, only (2n,0,l) and (2n,0,0) diffraction lines should be visible [12]. At approximately 2θ > 30°, diffraction lines from W_n_O_3n−1_ and m-WO_3_ overlap and thus cannot be used to determine the structure or the stoichiometry. 

The valence band spectrum shows some density of states at the Fermi energy. The near-Fermi bands are formed due to 5d- and W 6s-like states taking part in the formation of the shortened W-W bonds [34] or due to trap states created by defects [35]. This could indicate a slightly metallic behaviour at room temperature, instead of a semiconducting one. DFT calculation on similar stoichiometries shows that the 5d-orbitals of tungsten atoms, which are part of the CS planes, are responsible for the conductivity and other effects related to the states near the Fermi surface [17,36].

The Raman spectra of the nanotiles have peaks of similar shape and position to those from the literature [20]. The spectra taken at the polarisation along the *b* axis have fewer peaks in the lattice and bending mode region than when the polarisation is perpendicular to the *b* axis. Compared to some other Raman spectra of WO_3−x_ nanomaterials [24,37,38], our spectra show narrower peaks, pointing to a higher degree of crystallinity. When the laser power is increased, the nanotiles oxidize to m-WO_3_ [24,37].

Due to the intrinsic oxygen vacancies and formation of CS planes, the electronic and optical properties of tungsten suboxides differ from m-WO_3_. Such properties may provide an advantage in applications such as water splitting [39], near-infrared shielding [40], in anode materials for high-performance Li-ion batteries [41], field-effect-transistors [42], photocatalysis [43], and in-domain boundary engineering [44]. As it was shown [35], sub-stoichiometric WO_3−x_ nanosheets can be used as physisorption-based NO_2_ sensors. A slight difference in the stoichiometry can change the WO_3−x_ materials from a semiconductor to a metal, which can result in a poorer performance of such sensors. As pristine WO_3_ does not have a high photocatalytic activity, introducing oxygen vacancies and/or using lower dimensional WO_3−x_ can improve its performance. In several studies [45,46], the WO_3−x_ materials outperformed pristine WO_3_ in the degradation of dyes such as methylene blue, congo red, and rhodamine B. The oxygen vacancies act as electron donors, increasing the charge transport and thus enhancing the photocatalytic activity. Sub-stoichiometric materials also outperform m-WO_3_ when it comes to water splitting [47]. By annealing the samples under different atmospheres, the number and nature of oxygen vacancies were altered. It was concluded that the moderate concentration of oxygen vacancies results in appearance of W^5+^ shallow donor states that increase photoactivity, while the deep trap W^4+^ states have a detrimental effect on photocurrent. Being able to determine the stoichiometry and with it the electrical and optical properties of WO_3−x_ nanomaterials offers new opportunities for a wide range of applications.

## 5. Conclusions

Multi-stoichiometric nanotiles were synthesized using the CVT method. The thickness of the nanotiles ranged from a few 10 to a few 100 nm, and they grew up to a few µm in the lateral size. The formation of {102}_r_ CS planes indicates, that only W_n_O_3n−1_ phases grow inside the nanotiles. Three new stoichiometries were identified from HRTEM images: W_13_O_38_ (WO_2.923_), W_12_O_35_ (WO_2.917_), and W_11_O_32_ (WO_2.909_). Measured unit cell parameters agreed well with the calculated ones. The valence band spectrum showed some density of states at the Fermi energy, making the material slightly metallic. Obtained Raman spectra showed multiple peaks and are direct evidence of the material anisotropy. Increasing the laser power during Raman spectroscopy promoted the oxidation of the platelets into m-WO_3_.

## Figures and Tables

**Table 1:** Measured and calculated unit cell parameters for observed phases.

	Measured				Calculated			
Structure	a (Å)	b (Å)	c (Å)	β (°)	a (Å)	b (Å)	c (Å)	β (°)
W_11_O_32_	17.3	3.83	18.9	79.3	17.1	>3.83	19.2	72.2
W_12_O_35_	17.3	3.83	20.2	89.6	17.1	>3.83	20.0	87.9
W_13_O_38_	17.3	3.83	22.5	103.7	17.1	>3.83	22.1	101.5
W_14_O_41_	17.3	3.83	24.5	74.2	17.1	>3.83	24.6	72.0
W_15_O_43_	17.3	3.83	25.6	88.0	17.1	>3.83	25.2	84.4
W_16_O_47_	17.3	3.83	27.5	98.3	17.1	>3.83	26.9	95.7

**Table 2:** Measured XRD diffraction lines (positions in ° and Å) and their relative intensities compared with the calculated d values using parameters obtained from HRTEM images and the assigned (hkl) indices.

Measured			Theoretical W_14_O_41_	
2 Theta (°)	d (Å)	Rel. Int.	Assigned Index (hkl)	d (Å)
12.5	7.08	0.02	$\left(20\bar{3}\right)$	7.04
18.8	4.70	0.01	(005)	4.68
21.5	4.13	0.05	(403)	4.17
23.2	3.84	0.14	(010)	3.83
23.9	3.73	0.30	(110)	3.73
24.5	3.63	0.66	(112)	3.63
25.0	3.56	1.00	$\left(40\bar{2}\right)$	3.52
28.9	3.09	0.02	(407)	3.09
34.2	2.62	0.04	$\left(21\bar{5}\right)$	2.62
48.5	1.88	0.10	$\left(71\bar{2}\right)$	1.88
50.3	1.81	0.08	(322)	1.81
52.7	1.74	0.03	$\left(12\bar{5}\right)$	1.74
57.6	1.60	0.02	$\left(52\bar{2}\right)$	1.60

**Table 3:** Raman peak position and normalized intensity of the nanotiles and WO_3_.

Modes	Parallel		Perpendicular		WO_3_ Powder	
	Raman Shift	Int	Raman Shift	Int	Raman Shift	Int
					71	0.65
Lattice modes	136.5	0.26	136	0.14	135	0.29
			180	0.09	186.5	0.06
			232.5	Sh	241	0.03
W-O bending			271.5	0.19	273	0.41
	322.5	0.02	331.5	0.12	327	0.07
	341.5	0.03	367	0.05	350	0.02
					417	0.01
	426.5	0.01	428	0.02	437	0.01
W-O stretching	722	sh	702	0.17	716	0.49
	810	1.00	810	1.00	807	1.00

## Data Availability

The data presented in this study are available on request from the corresponding author.

## Supplementary Materials

The following are available online at https://www.mdpi.com/article/10.3390/nano11081985/s1, Figure S1: (a) An AFM image of a nanotile with a line profile showing its height; (b) Figure S2: TEM image along the [010] direction. The white arrows point along the CS planes. Figure S3: interatomic distance between tungsten atoms that are not part of the CS plane (red) and interatomic distance between tungsten atoms that are part of the CS plane, where the tungsten octahedra are joined by edges (green).
