# Supplementary material for: Synthesis and Characterization of Tungsten Suboxide WnO3n−1 Nanotiles
Source: Nanomaterials (Basel). 2021 Aug 2;11(8):1985. doi: 10.3390/nano11081985 (PMC8398204; doi:10.3390/nano11081985)
Supplement: Supplementary file 1 [file nanomaterials-11-01985-s001.zip › nanomaterials-1309295-SI.pdf]

nanomaterials
MDPI

Supplementary Material

Synthesis and Characterization of Tungsten Suboxide W_nO_{3n-1} Nanotiles

Luka Pirker [1,†], Bojana Višić [1,2,*,†], Janez Kovač [1], Srečo D. Škapin [1] and Maja Remškar [1,3]

1 Jožef Stefan Institute, Jamova Cesta 39, 1000 Ljubljana, Slovenia; luka.pirker@ijs.si (L.P.); janez.kovac@ijs.si (J.K.); sreco.skapin@ijs.si (S.D.Š.); maja.remskar@ijs.si (M.R.)
2 Institute of Physics Belgrade, University of Belgrade, Pregrevica 118, 11080 Belgrade, Serbia
3 Faculty for Mathematics and Physics, University of Ljubljana, Jadranska ulica 19, 1000 Ljubljana, Slovenia
* Correspondence: bojana.visic@ipb.ac.rs
† These authors contributed equally.

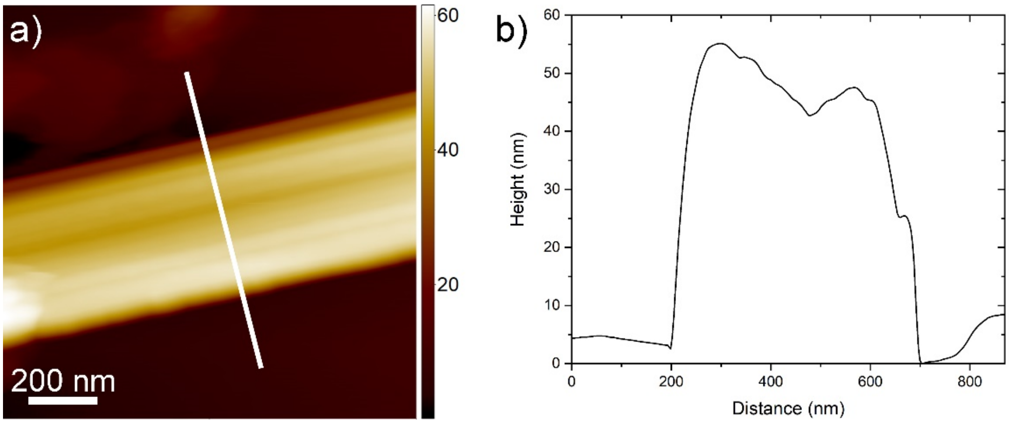

Figure S1. (**a**) An AFM image of a nanotile with a line profile showing its height (**b**).

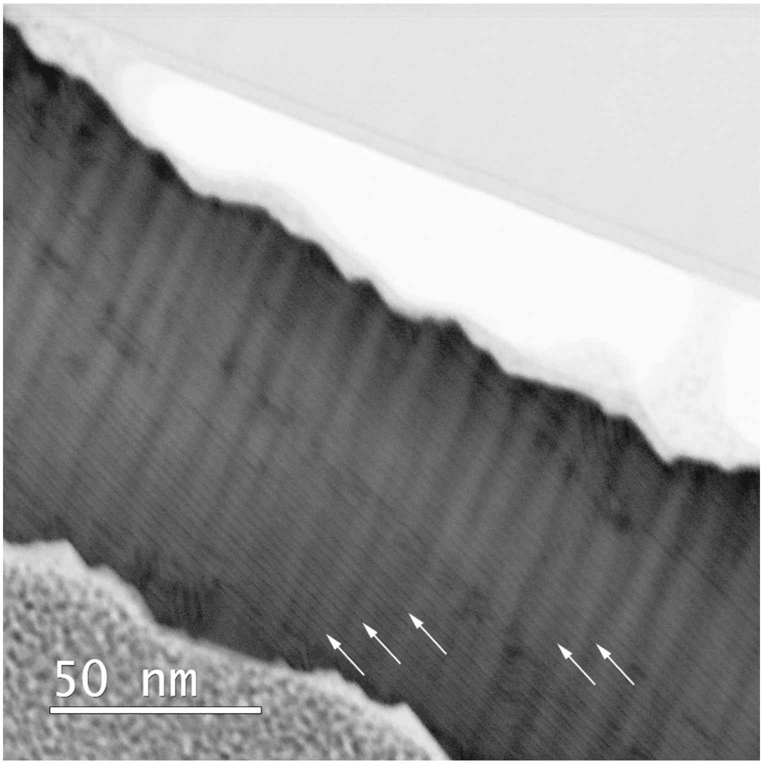

Figure S2. TEM image along the [010] direction. The white arrows point along the CS planes.

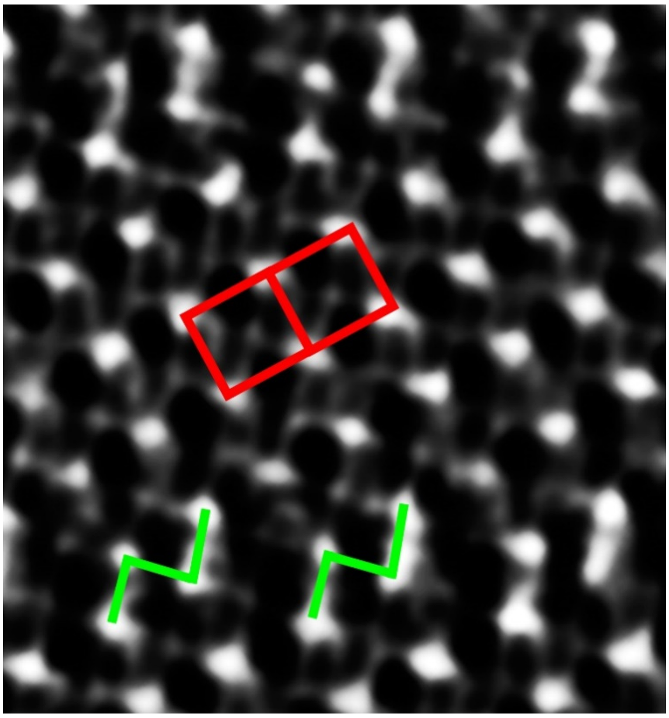

Figure S3. interatomic distance between tungsten atoms that are not part of the CS plane (red) and interatomic distance between tungsten atoms which are part of the CS plane, where the tungsten octahedra are joined by edges (green).
